# First-Principles Study on the Effect of Strain on Single-Layer Molybdenum Disulfide

**DOI:** 10.3390/nano11113127

**Published:** 2021-11-19

**Authors:** Chen Chong, Hongxia Liu, Shulong Wang, Kun Yang

**Affiliations:** School of Microelectronics, Xidian University, Xi’an 710071, China; 18829029042@163.com (C.C.); kuny2019@163.com (K.Y.)

**Keywords:** molybdenum disulfide (MoS_2_), tensile strain, compressive strain, energy band

## Abstract

By adopting the first-principles plane wave pseudopotential method based on density functional theory, the electronic structure properties of single-layer MoS_2_ (molybdenum disulfide) crystals under biaxial strain are studied. The calculation results in this paper show that when a small strain is applied to a single-layer MoS_2_, its band structure changes from a direct band gap to an indirect band gap. As the strain increases, the energy band still maintains the characteristics of the indirect band gap, and the band gap shows a linear downward trend. Through further analysis of the density of states, sub-orbital density of states, thermodynamic parameters and Raman spectroscopy, it revealed the variation of single-layer MoS_2_ with strain. This provides a theoretical basis for realizing the strain regulation of MoS_2_.

## 1. Introduction

Since the first use of mechanical exfoliation to obtain graphene films in 2004, the experimental significance of two-dimensional materials has been revealed [1]. With the deepening of the research on graphene, the shortcomings of graphene such as its low switching ratio and short carrier lifetime have affected the development of graphene. Therefore, transition metal sulfides have entered the field of vision of scholars as a new two-dimensional optoelectronic material [2]. Due to the excellent optical, electrical, lubricating, and catalytic properties of these transition metal layered two-dimensional compounds, they have attracted the research interest of a large number of researchers [3,4,5]. Among these compounds, MoS_2_ has excellent chemical and thermal stability, large specific surface area and high surface activity [6]. Due to the superiority of its structure, it has been widely studied for application in field effect transistors [7,8], optoelectronic devices [9,10], solid lubricants [11,12] and electrochemical hydrogen storage [13,14]. Therefore, researchers have conducted in-depth research on the preparation method, electronic structure characteristics, and optical characteristics of MoS_2_ [15,16]. At the same time, the effects of defects and other factors on MoS_2_ have also been studied [17,18].

Monolayer MoS_2_ is a direct bandgap semiconductor with a band gap of 1.8 eV [19,20]. Each monolayer MoS_2_ is composed of a layer of Mo atoms and two upper and lower layers of S atoms, and the distance between the layers is 6.15 Å [21,22]. Lee et al. studied the Raman spectra of MoS_2_ under different layers [23]. The experimental results show that the two Raman modes $E_{2g}^{1}$ and A_1g_ exhibit a strong thickness dependence. Xin He et al. studied the energy band changes of WS_2_ and MoS_2_ under strain through photoluminescence spectroscopy and first-principles calculations [24]. Wenbin Zhang used a linear displacement device to realize the strain of a two-dimensional material, thereby studying the effect of strain on its optical properties and Raman characteristic peaks [25].

The MoS_2_ bulk material is an indirect band gap, and the monolayer material is a direct band gap. This is the result of weak van der Waals forces between layers. Therefore, the single-layer MoS_2_ structure is easily affected by external factors. In this paper, through first-principles calculations, the physical mechanism of the formation of a single-layer MoS_2_ band structure will be analyzed, and the changes in its band structure, electronic structure, thermodynamic parameters and Raman spectra will be studied by applying stress.

## 2. Calculation Model and Method

There are many ways to apply strain in the experiment, mainly including stretching method and bending method. The stretching method mainly stretches the flexible substrate to achieve the purpose of applying strain to the material [26]. The most commonly used bending method is the three-point method—that is, the three-point fixed bending method applies strain to the material by bending the substrate [27].

The first-principles method in this paper was based on the CASTEP module of Materials Studio software (Materials Studio 2017, Accelrys, San Diego, CA, USA), which mainly uses density functional theory (DFT) to calculate and analyze the related properties of MoS_2_ [28]. The density functional between electrons and electrons was realized by the Perdew-Burke-Ernzerhof (PBE) algorithm under generalized gradient approximation (GGA) [29]. The density of states and structure optimization of all atomic orbitals were realized by the Broyden-Fletcher-Goldfarb-Shanno (BFGS) algorithm. At the same time, the plane wave truncation energy was set to 400 eV, and the atomic stress was less than 0.05 eV/Å. All this was done to provide the structure with better convergence. The relaxation convergence accuracy was 1 × 10^−5^ eV/atom.

**Figure 1:**
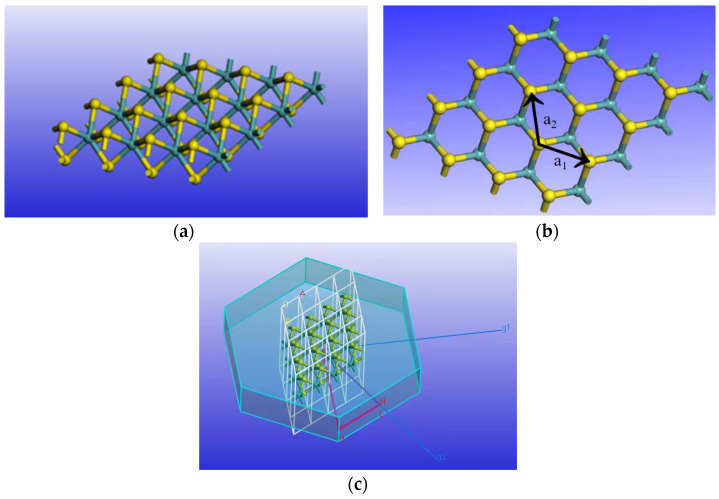
(**a**) Optimized MoS_2_ structure, (**b**) the direction of a_1_ and a_2_ in MoS_2_, and (**c**) the k-point grid in reciprocal space.

First, the single-layer unit cell structure was optimized. The optimized structure is shown in Figure 1. The bond length of Mo and S was 2.427 Å, the S—Mo—S bond angle was 82.276°, and the optimized lattice constant was 3.160 Å. This is consistent with the calculation result of A. Molina-Sanchez [30]. Then, tensile stress was applied in the directions of a_1_ and a_2_, respectively, and the resulting lattice strain (ε) was defined as the ratio of the change in the lattice constant (∆a) to the lattice constant (a).

(1) $$\varepsilon =\frac{\Delta a}{a}$$

This process was achieved by directly changing its lattice constant. After straining, all atoms in the unit cell were also fully relaxed by the same standard.

The corresponding lattice constants under different strains are shown in Table 1.

## 3. Results and Discussion

### 3.1. Energy Band

**Figure 2:**
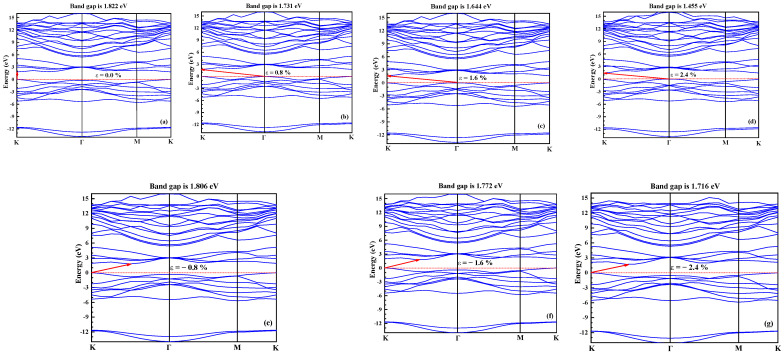
Band structure of single-layer MoS_2_ under different strains.

Figure 2 shows the energy band structure of a single layer of MoS_2_ under different tensile stresses. It can be seen from the energy band structure that when no stress is applied, the energy band of MoS_2_ is a direct band gap, with the highest point of the valence band and the lowest point of the conduction band, which are both K points. As shown in Figure 2a, the band gap is 1.822 eV, which is similar to the calculation result in Humberto Terrones [31]. When a tensile strain of 0.8% is applied in the layer, the lowest point of the conduction band is still at the K point, but the highest point of the valence band is transferred from the K point to the Γ point. The energy band of the single-layer MoS_2_ changes from the direct band gap to an indirect band gap. As shown in Figure 2, the band gap changes from 1.822 to 1.731 eV. As the tensile strain continues to increase, the band structure always maintains the characteristics of indirect band gap. When a compressive strain of 0.8% is applied to the layer, the lowest point of the valence band is still at point K, but the highest point of the conduction band is transferred from point K to the between the K and Γ points. The energy band of the single-layer MoS_2_ is changed from the direct band gap to the indirect band gap. It can be seen from Figure 2 that the band gap changes from 1.822 to 1.806 eV. As the compressive stress continues to increase, the band structure always maintains the characteristics of the indirect band gap.

**Figure 3:**
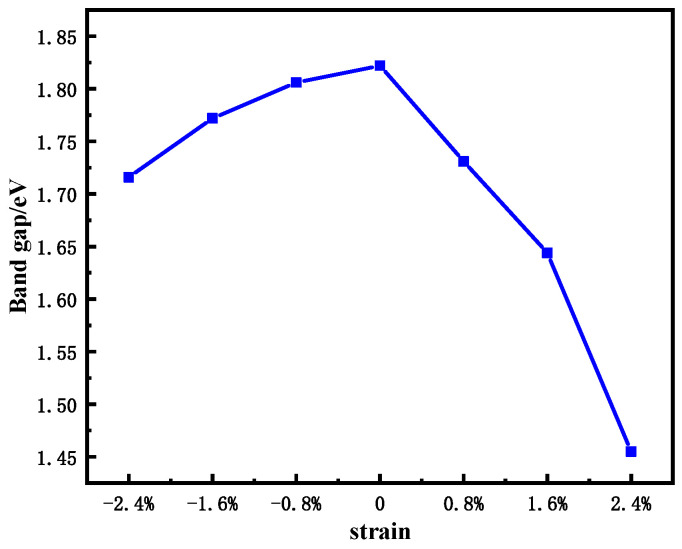
Band gap change of single-layer MoS_2_ under different strains.

As shown in Figure 3, as the strain increases (whether it is tensile strain or compressive strain), the band gap of the single-layer MoS_2_ becomes smaller. Under tensile strain, the band gap decreases much more than under compressive strain. This is because the distance between the bottom of the conduction band and the top of the valence band under compressive strain is much smaller than that under tensile strain.

### 3.2. Density of States

**Figure 4:**
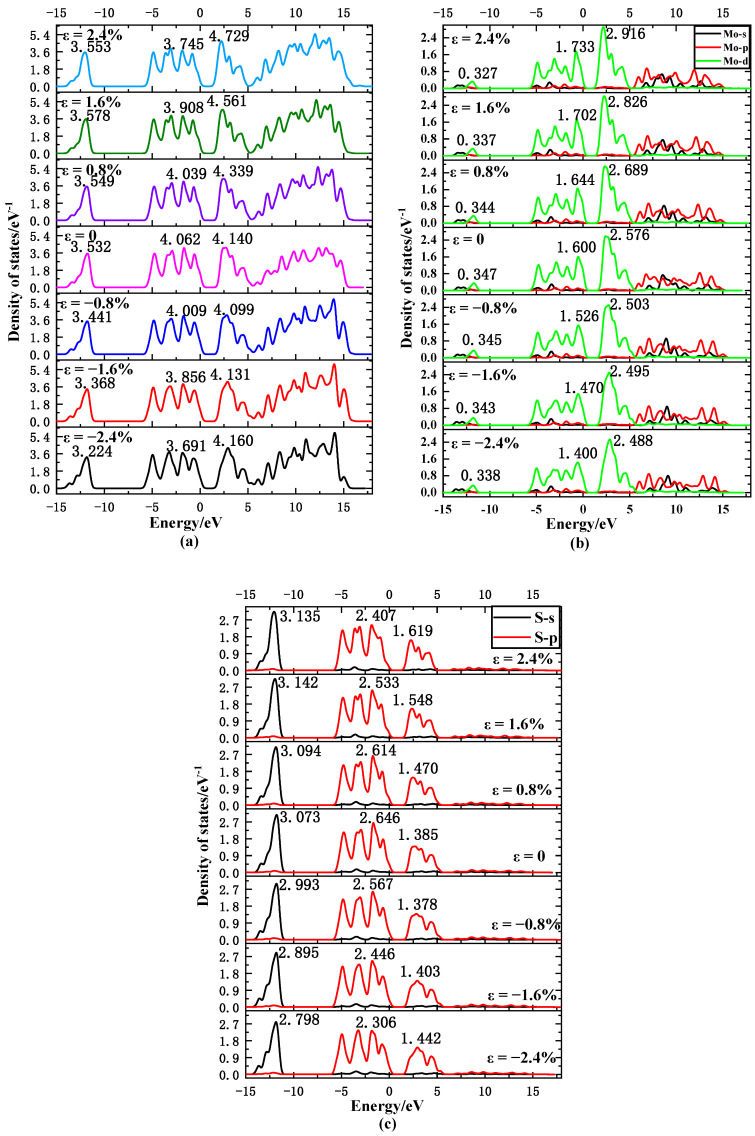
The total density of states of MoS_2_ (**a**), Mo (**b**), S (**c**) atomic suborbital state density changes with strain.

As shown in Figure 4, the density of states diagram of a single-layer MoS_2_ is mainly composed of four parts. Among them, the −14~−11 eV region is mainly composed of the s-orbital electrons of S atoms; in the −5~0 eV region, the shape of the Mo atom’s d orbital and the S atom’s p-orbital density of states is very similar, which indicates that the electrons are shared between the two, and the p-orbital peak of the S atom is higher. The contribution to the density of states is dominant, which corresponds to the d-orbital of the Mo atom and the p-orbital of the S atom, forming a weaker interaction similar to the π bond; in the range of 1~5 eV, it is also hybridized by the d-orbital electron of the Mo atom and the p-orbital electron of the S atom. However, the molybdenum atom’s d orbital peak is higher and the contribution to the density of states is dominant. This corresponds to the weaker π bond interaction formed by the Mo atom’s d orbital and the S atom’s p orbital. Studies have shown that this kind of π-like bond is highly sensitive to strain, which causes strain to affect its band structure. The 5~15 eV region is mainly composed of the electrons of the Mo atom’s s orbitals and p orbitals.

In this paper, peak A is the highest peak in the −14~−11 eV region, peak B is the highest peak in the −5~0 eV region, and peak C is the highest peak in the 1~5 eV region. It can be seen from Figure 4 that as the tensile strain increases, the peak state density of MoS_2_ at peak A increases and then decreases, which is completely consistent with the change trend of the peak state density of S atoms at peak A. This also proves that the s-orbital electrons of S atoms are mainly used in the −14~−11 eV region. It can be seen from Figure 4 that with the increase in tensile strain, the peak state density of MoS_2_ at peak B becomes smaller, which is consistent with the change trend of peak state density of S atom at peak B. This shows that the −5~0 eV region is mainly affected by the p-orbital electrons of the S atom. It can be seen from Figure 4 that as the tensile strain increases, the peak state density of MoS_2_ at the C peak becomes smaller, which is different from the change trend of the peak state density of S and Mo atoms at the C peak. Therefore, in the 1~5 eV region, it is composed of the d-orbital electron of Mo atom and the p-orbital electron of S atom. The peak B is close to the top of the valence band, and the energy of the electrons in the S 2p state is high, so that the interaction between the electrons in the S 2p state and the Mo 3d state is enhanced, and the position of the valence band is shifted upward. While the peak C is close to the bottom of the conduction band, the electron energy in the Mo 3d state is too high, causing the interaction between the Mo 3d state electrons and the S 2p state electrons to increase, and the conduction band position is shifted downward. Therefore, as the tensile strain increases, the band gap of MoS_2_ decreases.

It can be seen from Figure 4 that as the compressive strain increases, the peak state density of MoS_2_ at peak A also increases, which is consistent with the change trend of the peak state density of Mo and S atoms at peak A. The density of states of S atoms in the s orbital is much larger than that of Mo atoms in the d orbital, so it is mainly the electrons in the S 1s state that play a major role. It can be seen from Figure 4 that as the compressive strain increases, the peak state density of MoS_2_ at peak B becomes smaller and smaller, which is consistent with the change trend of the peak state density of Mo and S atoms at peak B. The density of states of S atoms in the p orbital is higher than that of Mo atoms in the d orbital, and so the S 2p state electrons play a major role. It can be seen from Figure 4 that as the compressive strain increases, the peak state density of MoS_2_ at the C peak first decreases and then increases, which is consistent with the change trend of the peak state density of the S atom at the C peak. The density of states of Mo atoms in the d orbital is higher than that of S atoms in the p orbital. Therefore, the total density of states of MoS_2_ is composed of the hybridization of the d orbital electrons of the Mo atom and the p-orbital electrons of the S atom. Since the C peak is close to the bottom of the conduction band, the total density of states of MoS_2_ increases. Therefore, the interaction between the Mo 3d state electrons and the S 2p state electrons is more enhanced. This means that the conduction band position is offset downwards under compressive strain. This also shows that, compared with tensile strain, the band gap of MoS_2_ becomes narrower under compressive strain.

### 3.3. Thermodynamic Properties and Raman Spectrum

The thermodynamic properties of MoS_2_ are calculated using the quasi-harmonic Debye model [32], and the temperature range and strain range are 0 to 1000 K and −2.4% to 2.4%, respectively. The thermodynamic parameters free energy *F*(*T*), entropy S and heat capacity *C_v_* can be obtained by the following formula [33]:

(2) $$E\left(T\right)=E_{total}+E_{zp}=\int \frac{hw}{\exp\left(\frac{hw}{kT}\right)-1}F\left(w\right)d\left(w\right)$$

*E_zp_* is the zero-point vibrational energy, *h* is Planck’s constant, *k* is Boltzmann’s constant, *F*(*ω*) is the phonon state density, and *E_zp_* can be defined as follows:

(3) $$E_{zp}=\frac{1}{2}\int F\left(w\right)hwddw$$

Free energy *F*(*T*):

(4) $$F\left(T\right)=E_{total}+E_{zp}+kT\int F\left(w\right)ln\left[1-\exp\left(-\frac{hw}{kT}\right)\right]dw$$

Entropy *S* is:

(5) $$S\left(T\right)=k\left\{\int \frac{\frac{hw}{kT}}{\exp\left(\frac{hw}{kT}\right)-1}F\left(w\right)d\left(w\right)-\int F\left(w\right)\left[1-\exp\left(-\frac{hw}{kT}\right)\right]dw\right\}$$

Heat capacity *C_v_* is:

(6) $$C_{v}\left(t\right)=k\int \frac{{\left(\frac{hw}{kT}\right)}^{2}\exp\left(\frac{hw}{kT}\right)}{{\left[\exp\left(\frac{hw}{kT}\right)-1\right]}^{2}}F\left(w\right)dw$$

**Figure 5:**
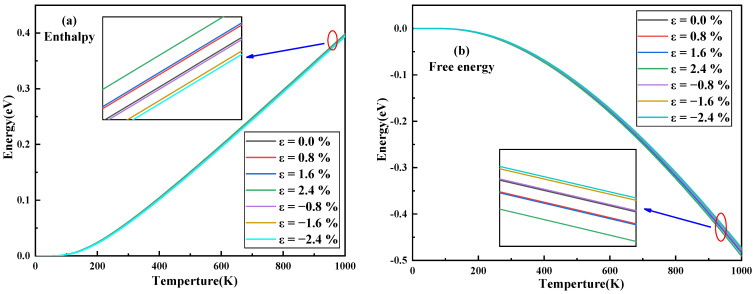

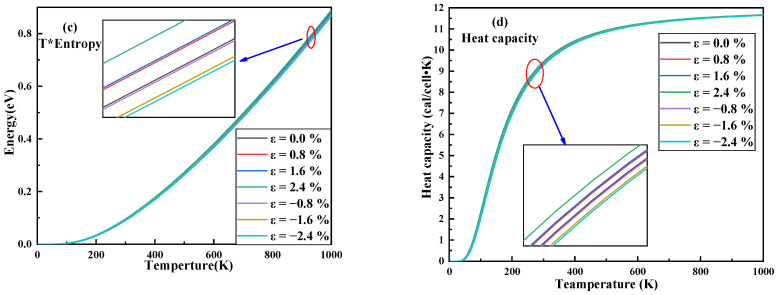
Enthalpy (**a**), free energy (**b**), entropy (**c**) and heat capacity (**d**) of a single layer of MoS_2_ under different strains.

Enthalpy is a parameter for judging the thermal vibration state of a material. It can be seen from Figure 5a that the enthalpy increases linearly with the increase in temperature. When the temperature is constant, the enthalpy increases with the increase in tensile strain and decreases with the increase in compressive strain. The change in free energy with strain is shown in Figure 5b. As the temperature increases, the free energy decreases. When the temperature is constant, the free energy decreases with the increase in tensile strain and increases with the increase in compressive strain. The effect of strain on entropy is shown in Figure 5c. Entropy increases exponentially with increasing temperature. When the temperature is constant, the entropy increases with the increase in tensile strain and decreases with the increase in compressive strain. The heat capacity *C_v_* is an important parameter to evaluate the thermodynamic properties of materials. The effect of strain on the heat capacity is shown in Figure 5d. When the temperature is lower than 400 K, the heat capacity increases exponentially with the increase in temperature. When the temperature is greater than 400 K, the Dulong-Petit limit is reached, and the heat capacity is almost constant. When the temperature is constant, the heat capacity increases with the increase in tensile strain and decreases with the increase in compressive strain.

The Debye temperature *θ* can be obtained according to the following equation [34,35]:

(7) $$\theta =\frac{h}{k}{\left[\frac{3n}{4\pi }\left(\frac{N_{A}\rho }{M}\right)\right]}^{\frac{1}{3}}V_{m}$$

(8) $$V_{m}={\left[\frac{1}{3}\left(\frac{1}{V_{s}^{3}}+\frac{1}{V_{l}^{3}}\right)\right]}^{-\frac{1}{3}}$$

(9) $$V_{l}=\sqrt{\frac{\left(B+\frac{4}{3}G\right)}{\rho }}$$

(10) $$V_{s}=\sqrt{\frac{G}{\rho }}$$

**Figure 6:**
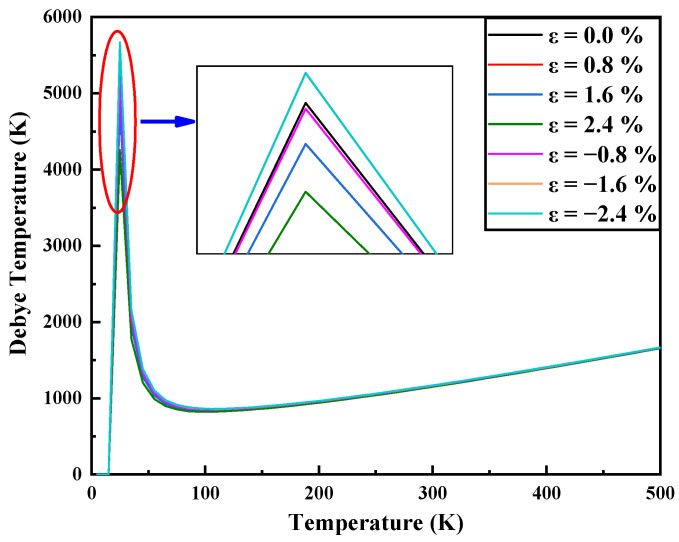
Debye temperature of a single layer of MoS_2_ under different strains.

The Debye temperature *θ* can reflect the chemical bond strength, structural stability, lattice vibration and changes in specific heat. It is closely related to many thermodynamic properties. It can be seen from Figure 6 that as the compressive strain increases, the Debye temperature also increases. With the increase in tensile strain, the Debye temperature decreases. This shows that applying compressive strain can enhance the mechanical and thermodynamic stability of MoS_2_.

**Figure 7:**
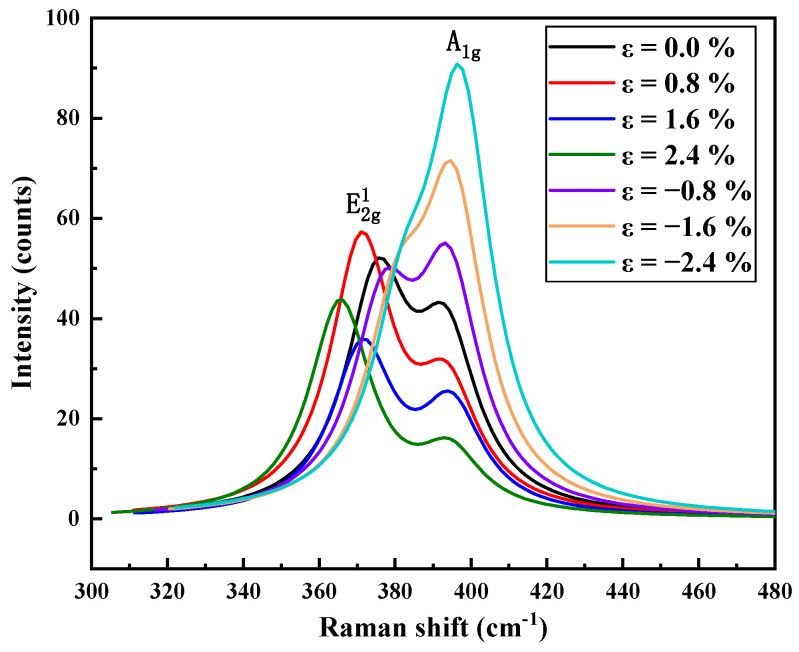
Raman spectrum distribution of a single layer of MoS_2_ under tensile strain and compressive strain.

Raman spectroscopy is a mature technique for studying the strain of molybdenum disulfide. By observing the phonon mode of the material using Raman spectroscopy, the effect of strain can be detected. As shown in Figure 7, the Raman spectrum of MoS_2_ is composed of two peaks, $E_{2g}^{1}$ and A_1g_. The difference between the abscissas of peak $E_{2g}^{1}$ and peak A_1g_ is 18 cm^−3^, which is the same as the experimental data of single-layer MoS_2_ in Han’s paper [7]. The $E_{2g}^{1}$ peak corresponds to the vibration of the Mo and S atoms of the layer, which is characteristic of the in-plane lattice vibration. The A_1g_ peak corresponds to the vibration of S atoms perpendicular to the layer, which is characteristic of out-of-plane lattice vibration. With the increase in tensile strain, the peak value of A_1g_ gradually decreases and is slightly red-shifted, and with the increase in compressive strain, the peak value of A_1g_ gradually increases and is slightly blue-shifted, which means that the vibration of S atoms is regularly affected by the strain.

## 4. Conclusions

In this paper, tensile strain and compressive strain are applied to a single layer of MoS_2_ by changing the lattice constant. The simulation results show that as the strain increases, the band gap of MoS_2_ narrows. Additionally, under compressive strain, the degree of band gap narrowing is much higher than that of tensile strain. At the same time, the thermodynamic properties of MoS_2_ under compressive strain are more stable by researching the thermodynamic parameters. Finally, by studying the changes in the Raman spectra under different strains, it can be obtained that the effect of strain on the vibration of the S atom of MoS_2_ is regular. All these findings provide research directions for the future research into MoS_2_ strain.

## Figures and Tables

**Table 1:** Lattice constants at different strains.

Strain	−2.4%	−1.6%	−0.8%	0	0.8%	1.6%	2.4%
Lattice constant/Å	3.08416	3.10944	3.13472	3.160	3.18528	3.21056	3.23584
